# The Low Fall as a Surrogate Marker of Frailty Predicts Long-Term Mortality in Older Trauma Patients

**DOI:** 10.1371/journal.pone.0137127

**Published:** 2015-09-01

**Authors:** Ting Hway Wong, Hai V. Nguyen, Ming Terk Chiu, Khuan Yew Chow, Marcus Eng Hock Ong, Gek Hsiang Lim, Nivedita Vikas Nadkarni, Dianne Carrol Tan Bautista, Jolene Yu Xuan Cheng, Lynette Mee Ann Loo, Dennis Chuen Chai Seow

**Affiliations:** 1 Singapore General Hospital, Singapore, Singapore; 2 Duke-National University of Singapore, Singapore, Singapore; 3 Tan Tock Seng Hospital, Singapore, Singapore; 4 Health Promotion Board, Singapore, Singapore; 5 National University Hospital System, Singapore, Singapore; University of New South Wales, AUSTRALIA

## Abstract

**Background:**

Frailty is associated with adverse outcomes including disability, mortality and risk of falls. Trauma registries capture a broad range of injuries. However, frail patients who fall comprise a large proportion of the injuries occurring in ageing populations and are likely to have different outcomes compared to non-frail injured patients. The effect of frail fallers on mortality is under-explored but potentially significant. Currently, many trauma registries define low falls as less than three metres, a height that is likely to include non-frailty falls. We hypothesized that the low fall from less than 0.5 metres, including same-level falls, is a surrogate marker of frailty and predicts long-term mortality in older trauma patients.

**Methods:**

Using data from the Singapore National Trauma Registry, 2011–2013, matched till September 2014 to the death registry, we analysed adults aged over 45 admitted via the emergency department in public hospitals sustaining blunt injuries with an injury severity score (ISS) of 9 or more, excluding isolated hip fractures from same-level falls in the over 65. Patients injured by a low fall were compared to patients injured by high fall and other blunt mechanisms. Logistic regression was used to analyze 12-month mortality, controlling for mechanism of injury, ISS, revised trauma score (RTS), co-morbidities, gender, age and age-gender interaction. Different low fall height definitions, adjusting for injury regions, and analyzing the entire adult cohort were used in sensitivity analyses and did not change our findings.

**Results:**

Of the 8111 adults in our cohort, patients who suffered low falls were more likely to die of causes unrelated to their injuries (p<0.001), compared to other blunt trauma and higher fall heights. They were at higher risk of 12-month mortality (OR 1.75, 95% CI 1.18–2.58, p = 0.005), independent of ISS, RTS, age, gender, age-gender interaction and co-morbidities. Falls that were higher than 0.5m did not show this pattern. Males were at higher risk of mortality after low falls. The effect of age on mortality started at age 55 for males, and age 70 for females, and the difference was attributable to the additional mortality in male low-fallers.

**Conclusions:**

The low fall mechanism can optimize prediction of long-term mortality after moderate and severe injury, and may be a surrogate marker of frailty, complementing broader-based studies on aging.

## Introduction

Trauma in older patients has emerged as an important multi-disciplinary issue, not just for the surgical disciplines but also for the geriatric, rehabilitation, intensive care, emergency medicine and pre-hospital specialties [1–5]. Predicting health outcomes in these patients is important for clinical benchmarking as well as formulating health policy. Trauma in older adults differs from young adult trauma in its epidemiology[6–8], resource utilization[9–11], risk factors for morbidity and mortality[12, 13], performance on injury outcome predictors[14, 15], and volume-outcome relationships[16–18].

Frailty is also a growing public health issue in ageing populations. The incidence of frailty increases with age, from just 3.2% for the age group 65–70 to 25.7% for the age group 85–89[19]. Frail elderly, when compared to the non-frail, have been found to have a higher rate of falls, hospitalization, functional decline and mortality.

Injury severity scores alone are poor predictors of mortality in older trauma patients[20]. Other factors specific to older adults need to be accounted for, such as frailty[21] and comorbidities[14, 15, 22–24]. The incorporation of co-morbidities can improve prediction of mortality after injury[14, 15, 22]. Co-morbidities, however, do not represent all that is known about frailty[25, 26]. Frailty has been shown to predict mortality independent of injury severity scores in prospective studies[21]. However, pre-injury frailty data are usually not available in trauma registry data, which are collected at the time of presentation with injury. Post-injury tests of frailty may be affected by injury and subsequent disability. In a study on injured elderly patients in intensive care, radiologically-diagnosed low skeletal muscle mass was significantly more common in falls and low-energy patients[27]. As injuries arising from lower fall heights are more likely to be frailty-related than those from higher fall heights, the low fall is a potential marker of frailty in trauma registry data and is expected to be a predictor of mortality. While the full spectrum of frailty cannot be explored from the information routinely captured in trauma databases, we propose that low falls (including same-level falls) could be a surrogate marker of frailty and explains some of the excess mortality seen in older trauma patients.

Our hypothesis is that the low fall is associated with increased long-term mortality in elderly trauma patients independent of age, gender, co-morbidities and injury severity scores. Data on mechanism of injury is routinely collected in trauma registries and diagnosis codes[28]. Studies have examined the effect of sub-categories of mechanism of injury on outcomes[29], as well as the relative increased mortality from same-level falls compared to higher level falls[30]. We set out to build on these studies by controlling for injury severity and co-morbidities. Exact fall heights are difficult to extrapolate from patient histories, and consensus or expert definitions of “same-level fall” or “low fall”[31] are not easily standardized for data entry teams. In view of these difficulties, many large registries either do not differentiate fall heights, or define low falls as less than three metres [32, 33], a height that is likely to include non-frailty falls, and can be life-threatening for young adults as well. For our registry, we implemented a standardized conversion table (furniture, steps, buildings) for converting patient histories to fall heights (Table 1). Using this data, we set out to define which low fall height optimally predicts mortality, using a standardized conversion methodology for patient histories. Higher energy injury is usually associated with a higher risk of mortality, which is why penetrating injury is treated differently from blunt trauma in injury severity scoring systems[34, 35]. Hence, if our hypothesis holds true that the low-energy mechanism is paradoxically associated with a higher risk of death and complications, then this could be because such patients are more frail than patients with similar injury patterns after high energy trauma.

**Table 1:** Guidelines for Conversion of Patient Histories to Heights in Metres.

Object	Height (metres)
Low stool or chair	0.50
High stool	1.00
Table	1.00
Single-deck bed	0.50
Double-deck bed	1.50
Each step of stairs	0.15
From car or passenger bus	0.50
From lorry	1.00
One storey	3.00

## Methods

### Data Source and Data Collection

Data are from the Singapore National Trauma Registry (NTR), established in 2011[8].

All hospitals that receive Singapore Civil Defence Force (SCDF) ambulances (i.e., all public hospitals) provide data for the NTR^42^. Data collection for the NTR is performed by teams of trained trauma data coordinators based at each public hospital, with data cleaning, data completeness, data quality and inter-rater audits performed annually. Quarterly reviews of data capture problems are performed by a central pool based at the National Registry of Diseases Office (NRDO). Patients meet inclusion criteria for the NTR if they present to hospitals’ emergency departments with any injury with the diagnostic codes 800–959.9, defined in the International Classification of Diseases, 9th Revision, Clinical Modification, excluding late effects of injury (codes 905–909.9)^43^. Once a trauma patient is identified by its ICD9 code in the emergency department, data coding personnel will check that the patient meets the inclusion criteria. Some fields are then automatically included in the registry by data capture from electronic medical records and all fields are checked by data coding personnel. Personnel are trained in coding of the Abbreviated Injury Scale (AIS), version 2005 update 2008^38^, capturing the physiological and anatomical variables from medical records for the calculation of the RTS and ISS respectively. Death date and cause of death are captured at quarterly intervals from the registry of births and deaths, provided by the National Registry of Diseases Office.

### Study Design

Retrospective data from January 2011 to December 2013 was matched with death registry data up to September 2014. The association between low falls and mortality (12-month and in-hospital) for patients aged over 45 was examined, controlling for injury severity score (ISS)[36], revised trauma score (RTS)[37], co-morbidities prevalent in 5% or more of the study population, Charlson co-morbidity index (CCI) [38], race (as entered by data entry personnel), gender, age and the age by gender interaction.

The effect of low fall on long-term survival and cause of death were explored by age-group and gender. Expanded sets of control variables were used in sensitivity analyses including residency status, pattern of injury (Abbreviated Injury Scale (AIS) score of three or more in each AIS region[39]), polytrauma [40, 41], and co-morbidities prevalent in 1% or more of the study population known to be associated with fall risk, worse outcomes after trauma or frailty (history of previous injury, visual defects, anaemia, dementia, depression and other psychiatric illness)[14, 42].

### Study Population

Study population included patients age 45 and over, as the low falls in the age-group 18 to 45 constituted less than one percent of all blunt trauma in this age-group. For patients with ISS less than 9 and patients aged 65 and over with isolated hip fractures (neck of femur or inter-trochanteric) sustained in same-level falls, detailed co-morbidity data, ISS and RTS were not available in the NTR. These patients were excluded from our analysis.

### Outcome measures

The primary outcome measure was mortality 12 months post-injury. Secondary outcome measures were long-term survival (death at any point during from the study period up to September 2014) and in-hospital mortality (primary admission for the injury). Cause of death was considered trauma-related if the death certificate showed any trauma-related diagnosis in the primary or secondary causes of death, and as non-trauma-related if there was no mention of trauma.

### Covariates

#### Mechanism of Injury

In the registry, injury mechanism is coded into the following categories: motor vehicle accident, fall, interpersonal violence, machinery, tools / objects, sports, unknown and others. Additional mechanism of injury details are available for motor vehicle injuries (vehicle, motorcycle, pedestrian, cyclist) and falls (height of fall). Burns and penetrating injuries were excluded from analysis.

#### Defining Low Falls

Falls in the registry are sub-divided into same-level falls and falls with height documented. The definition of same-level fall was set as falls from 0.5 m or less, to include falls from sitting or lying positions. Some patients in the category of falls with height documented had fall heights of 0.5 m or less, and were included in this final definition of low fall. Furniture, step and storey heights were defined based on consensus and building guidelines to convert patient histories (Table 1).

We compared low falls as a blunt mechanism subtype to falls from other heights, and to other blunt mechanism sub-types, including motor vehicle injuries. Sensitivity analyses included sub-dividing the motor vehicle injuries into the commonest sub-types (car, motorcycle, pedestrian) and using different cut-offs for low falls (1m, 2m, and 3m)[32–34].

#### Age

Age was analyzed in ten-year bands from age 45, the highest age band as age 85 and over. The 65–74 year age band was split into 5-year bands, to include many commonly used age cut-offs in the literature[34, 35, 41, 43–45]. The referent age band was the 45–54 year band.

#### Measures of Injury Severity and Comorbidity

ISS and RTS were the anatomical and physiological measures of injury severity respectively.

Co-morbidity was alternately measured by: 1. CCI; 2. comorbid conditions that contributed more than 5% (by frequency) to co-morbidities found in our study population (diabetes, hypertension, hyperlipidaemia and cancer); 3. as in (2), plus comorbid conditions that contributed 1–5% (by frequency) and known to be associated with fall risks or frailty[42] (sensitivity analysis).

#### Statistical Analysis

Patient characteristics at baseline were summarized by mean (standard deviation) or median (inter-quartile range) or frequency (%) as appropriate. Chi-square tests and Fisher’s exact test were performed to evaluate associations between the outcomes of interest and other categorical predictors of interest. Univariate logistic regression was used to analyze mortality (12-month and in-hospital). The predictors which were significant (p<0.05) in the univariate regression were entered into the multivariable regression. Variables which were not statistically significant but were clinically meaningful were retained in the multivariable model. Stata 13.0 was used.

Due to the age-gender interactions, sub-group analyses by gender were performed. Relative performance of prediction models was assessed using the Hosmer-Lemeshow goodness-of-fit test and c-statistic (i.e. area under the receiver operating characteristic curve ROC). The ROC was generated based on the final model to visualize sensitivity and specificity of the chosen model. Likelihood ratio tests were carried out to assess predictor significance by comparison between nested models. Long-term survival generated using Kaplan-Meier compared low fallers and non-low fallers using the log rank test.

Patients with missing data were omitted from the analysis: ISS (<0.5% missing), RTS (<0.5% missing), co-morbidities (missing 1.85%) and fall height (unknown 1.31%).

The first author’s (Singapore General Hospital) Institutional Review Board granted ethical approval for this retrospective study, as required prior to gaining access to the NTR data, which is de-identified prior to release for research, password-protected and access limited to the premises of the National Registry of Diseases Office (NRDO). Consent was not obtained because information was anonymized and de-identified prior to analysis, as per NRDO protocol.

## Results

### Descriptive analysis

In the three years of data collection 2011 to 2013, there were 8111 cases of blunt trauma patients meeting our study inclusion criteria. Summary statistics are presented in Table 2. Patients with low fall mostly suffered injuries to the head (48.1% AIS scorei3), extremities (37.2%), and torso (8.4% thoracic, 6.4% abdominal), with 2.7% having an AIS score of 3 or more in 2 or more body regions[40, 41]. The ISS ranged from 9 to 75. The AIS scores for all the body regions ranged from 0 (no injury) to 6 (unsurvivable injury), except for the “external” region (0 to 3) as isolated burns patients were excluded from this study.

**Table 2:** Characteristics of National Trauma Registry Patients aged over 45 (n = 8111).

	All	Age 45–54	Age 55–64	Age 65–69	Age 70–74	Age 75–84	Age 85 and above
Study Population	8111 (100)	1284 (15.8)	1879 (23.2)	764 (9.4)	854 (10.5)	1990 (24.5)	1340 (16.5)
Male	4417 (54.4)	1031 (80.3)	1246 (66.3)	450 (58.9)	446 (52.2)	839 (42.2)	405 (30.2)
Age mean (SD)	69.9 (13.6)	49.7 (2.9)	59.8 (2.9)	66.8 (1.5)	72.1 (1.4)	79.5 (2.9)	89.6 (3.9)
ISS mean (SD)	14.4 (8.0)	15.4 (9.5)	14.3 (8.6)	14.6 (8.4)	14.3 (7.4)	14.0 (7.1)	14.0 (6.9)
Head and Neck AIS ≥3	3727 (45.9)	524 (40.8)	751 (40.0)	379 (49.6)	449 (52.6)	967 (48.6)	657 (49.0)
Face AIS ≥3	33 (0.4)	11 (0.9)	12 (0.6)	5 (0.6)	2 (0.2)	3 (0.2)	0 (0.0)
Thorax AIS ≥3	1357 (16.7)	358 (27.9)	374 (19.9)	119 (15.6)	110 (12.9)	252 (12.7)	144 (10.7)
Abdomen AIS ≥3	586 (7.2)	123 (9.6)	121 (6.4)	50 (6.5)	50 (5.9)	137 (6.9)	105 (7.8)
Extremity AIS ≥3	2826 (34.8)	401 (31.2)	756 (40.2)	244 (31.9)	266 (31.1)	689 (34.6)	470 (35.1)
External AIS ≥3	4 (<0.1)	2 (0.2)	2 (0.1)	0 (0.0)	0 (0.0)	0 (0.0)	0 (0.0)
Polytrauma	615 (7.6)	169 (13.2)	168 (8.9)	52 (6.8)	46 (5.4)	100 (5.0)	80 (6.0)
RTS mean (SD)	7.5 (1.1)	7.3 (1.5)	7.5 (1.2)	7.5 (1.1)	7.6 (1.0)	7.6 (0.8)	7.6 (0.7)
Injury mechanism							
Road Traffic Injury (all)	1581 (19.5)	545 (42.4)	560 (29.8)	168 (22.0)	149 (17.4)	132 (6.6)	27 (2.0)
Motor Vehicle	278 (3.4)	117 (9.1)	96 (5.1)	30 (3.9)	19 (2.2)	13 (0.7)	3 (0.2)
Motorcycle	726 (9.0)	297 (23.1)	291 (15.5)	72 (9.4)	37 (4.3)	27 (1.4)	2 (0.1)
Pedestrian	221 (2.7)	56 (4.3)	72 (3.8)	32 (4.2)	38 (4.4)	21 (1.1)	2 (0.1)
Other Road Traffic Injury	356 (4.4)	75 (5.9)	101 (5.4)	34 (4.5)	55 (6.4)	71 (3.6)	20 (1.6)
All fall	6184 (76.2)	606 (47.2)	1205 (64.1)	567 (74.2)	676 (79.2)	1830 (92.0)	1300 (97.0)
Low fall (0–0.5m, inclusive)	5306 (65.4)	376 (29.3)	966 (51.4)	492 (64.4)	611 (71.5)	1671 (84.0)	1190 (88.8)
Fall > 0.5–1 metre	148 (1.8)	38 (3.0)	48 (2.6)	10 (1.3)	14 (1.6)	25 (1.3)	13 (1.0)
Fall > 1 to 2 metres	211 (2.6)	55 (4.3)	74 (3.9)	17 (2.2)	22 (2.6)	28 (1.4)	15 (1.1)
Fall >2 to 3 metres	196 (2.4)	43 (3.3)	67 (3.6)	16 (2.1)	9 (1.0)	35 (1.8)	26 (1.9)
Fall from >3 metres	262 (3.2)	87 (6.8)	64 (3.4)	20 (2.6)	17 (2.0)	34 (1.7)	40 (3.0)
Fall from unknown height	139 (1.7)	16 (1.2)	31 (1.6)	8 (1.0)	12 (1.4)	32 (1.6)	40 (3.0)
Assault	78 (1.0)	34 (2.6)	22 (1.2)	7 (0.9)	8 (0.9)	7 (0.4)	0 (0.0)
Blunt-other	268 (3.3)	99 (7.7)	92 (4.9)	22 (2.9)	21 (3.4)	21 (1.1)	4 (1.0)
Charlson Comorbidity Index (CCI) score 1	1804 (22.2)	136 (10.6)	302 (16.1)	180 (23.6)	217 (25.4)	591 (29.7)	374 (27.9)
CCI 2	665 (8.2)	31 (2.4)	98 (5.2)	56 (7.3)	74 (8.7)	218 (11.0)	185 (13.8)
CCI 3	226 (2.8)	13 (1.0)	44 (2.3)	18 (2.4)	26 (3.1)	78 (3.9)	45 (3.4)
CCI 4 and above	102 (1.3)	2 (0.2)	10 (0.5)	13 (1.7)	12 (1.4)	33 (1.7)	31 (2.3)
Mortality at hospital discharge	721 (8.9)	98 (7.6)	144 (7.7)	65 (8.5)	67 (7.8)	192 (9.6)	155 (11.6)
Mortality at 1-yr post-injury	1350 (16.6)	120 (9.3)	211 (11.2)	112 (14.7)	123 (14.4)	392 (19.7)	392 (29.3)
Resident	7454 (91.9)	999 (77.8)	1731 (92.1)	720 (94.2)	793 (92.9)	1908 (95.9)	1303 (97.2)
Chinese	6464 (79.7)	834 (65.0)	1438 (76.5)	616 (80.6)	708 (82.9)	1675 (84.2)	1193 (89.0)
Malay	791 (9.8)	180 (14.0)	201 (10.7)	78 (10.2)	75 (8.8)	178 (8.9)	79 (5.9)
Indian	569 (7.0)	170 (13.2)	169 (9.0)	43 (5.6)	53 (6.2)	94 (4.7)	40 (3.0)

*Data provided as N (%) unless otherwise noted. SD: standard deviation

### Multivariate Analysis of Age, Gender, ISS, RTS, Co-morbidities and Low Fall

The low fall was found to be an independent predictor of one-year mortality compared to higher levels of fall and other blunt mechanisms of injury after adjusting for age, gender, age by gender interaction, CCI, ISS and RTS (Table 3). The c-statistic of 0.8279 compared favorably with the model of the same covariates without low falls (0.8173), without age-gender interaction (0.8205), the model with age, gender, ISS, RTS and CCI (0.8169), and the model with only age, gender, ISS and RTS (0.8053).

**Table 3:** Logistic Regression analysis of 12-month mortality.

Covariates	Overall			Male			Female		
	OR	95% CI	p-value	OR	95% CI	p-value	OR	95% CI	p-value
Low Fall	1.75	1.18–2.58	0.005	2.03	1.37–3.02	<0.001	1.08	0.64–1.81	0.781
Non Low Fall	1.075	0.52–2.22	0.846	1.22	0.67–2.23	0.519	0.90	0.59–1.40	0.650
Male	0.72	0.33–1.57	0.406	*	*	*	*	*	*
ISS	1.07	1.07–1.08	<0.001	1.07	1.06–1.08	<0.001	1.07	1.06–1.08	<0.001
RTS	0.43	0.41–0.46	<0.001	0.43	0.40–0.46	<0.001	0.43	0.32–0.56	<0.001
Charlson Comorbidity Index (CCI) score 1	1.23	1.09–1.38	0.001	1.38	1.31–1.46	<0.001	1.03	0.76–1.41	0.844
CCI 2	2.18	1.57–3.03	<0.001	2.15	1.71–2.69	<0.001	2.34	1.51–3.65	<0.001
CCI 3	3.251	2.30–4.58	<0.001	3.70	2.20–6.15	<0.001	3.07	1.96–4.82	<0.001
CCI 4 and above	5.53	3.70–8.25	<0.001	5.80	3.78–8.90	<0.001	5.16	2.19–12.18	<0.001
Age 55–64 * Male	1.73	0.61–4.86	0.302	*	*	*	*	*	*
Age 65–69 * Male	2.05	0.78–5.38	0.147	*	*	*	*	*	*
Age 70–74 * Male	2.56	1.35–4.85	0.004	*	*	*	*	*	*
Age 75–84 * Male	2.49	1.26–4.90	0.008	*	*	*	*	*	*
Age 85 and above * Male	2.13	0.88–5.15	0.092	*	*	*	*	*	*
Age 55–64	1.29	0.59–2.83	0.526	2.11	1.21–3.66	0.008	1.47	0.64–3.41	0.363
Age 65–69	1.64	0.76–3.53	0.204	3.10	2.03–4.72	<0.001	2.07	0.92–4.68	0.079
Age 70–74	1.46	1.14–1.88	0.003	3.39	1.95–5.91	<0.001	1.88	1.36–2.60	<0.001
Age 75–84	2.41	1.51–3.86	<0.001	5.26	3.25–8.52	<0.001	3.91	1.82–6.11	<0.001
Age 85 and above	5.06	3.01–8.52	<0.001	9.66	5.30–17.59	<0.001	7.06	3.85–13.00	<0.001
Chinese	0.91	0.57–1.45	0.681	1.14	0.63–2.05	0.662	0.75	0.44–1.25	0.268
Malay	1.33	0.69–2.56	0.396	1.56	0.68–3.96	0.291	1.17	0.71–1.93	0.525
Indian	0.91	0.60–1.36	0.616	0.81	0.42–1.58	0.537	1.23	0.63–2.43	0.542
Constant	7.141	2.51–20.30	<0.001	4.06	1.13–14.54	0.031	12.12	2.86–51.32	0.001

*not applicable

Subgroup analysis by gender (Table 3) showed that the effect of increasing age on one-year and in-hospital mortality started at age 55 for males and 70 for females. Low falls were predictive of one-year mortality for males but not for females.

For in-hospital mortality, although the effect of low falls were not statistically significant after controlling for age, gender, co-morbidities, ISS and RTS, the odds of death in males were more than 30% higher, compared to other fall heights and other blunt injuries. Females were not at higher risk of mortality.

Some authors have explored the contribution of individual comorbidities to mortality after trauma[14], instead of using the CCI. For example, diabetes has been found to predict mortality in trauma patients independent of injury severity [46]. In our study, when the CCI was replaced by the co-morbidities that were prevalent in 5% or more of the study population, the low fall was still a significant predictor of twelve-month mortality (OR 1.88, 95% CI 1.24–2.86, p = 0.003) and remained significant in the sub-group analysis for males.

### Long-term Survival

**Fig 1:**
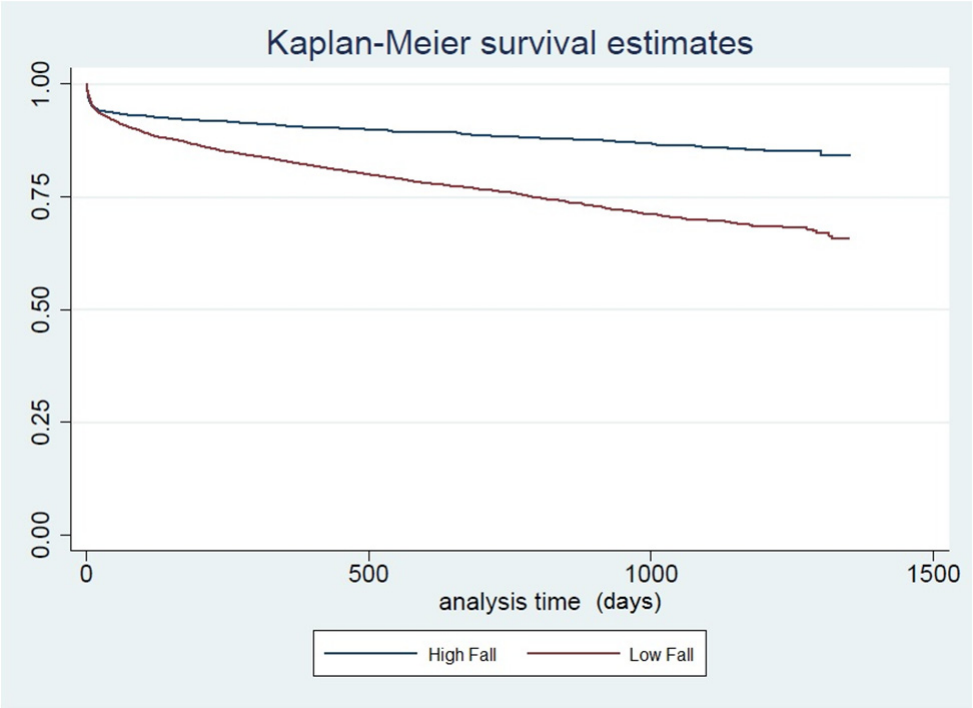
Survival Curve, Male, Low Fall vs High Falls.

**Fig 2:**
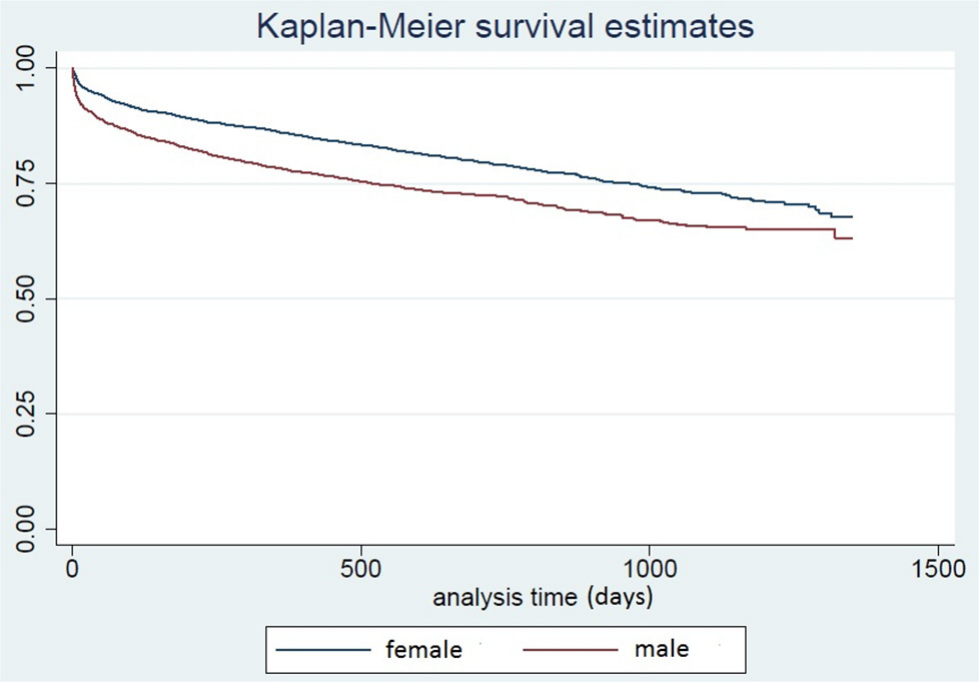
Survival Curve, Low Falls, Male vs Female.

Long-term survival curves comparing low falls vs higher falls for males, and male vs female patients after low fall are shown in Figs 1 and 2 respectively. These were significantly different based on the log rank test (p<0.001). For higher fall and other blunt trauma patients, there was no gender difference in long-term survival. The effect of low fall on increased mortality went beyond 12 months, the time-frame that most trauma studies use to study the out-of-hospital impact of trauma on mortality. For males, the effect of low fall on increased mortality was significant based on the log rank test for each age-band and hence was not due to the higher age of low fallers alone. The gender differences after low falls peaked in the first half of the study period, and declined towards the end of the study period. We did not consider time to event analysis because of the high number of in-hospital deaths.

### Cause of Death

Studies of gender differences for trauma have attributed excess mortality to the increased risk of infection in males after injury[47, 48]. In our population, the certified cause of death for low fall patients was less likely to be trauma-related (defined as any mention of injury in the primary or secondary causes of death) than for other mechanisms of blunt trauma in the same age group, and was the case for both genders and all ages (Table 4).

**Table 4:** Cause of Death for Patients with Mortality within 12 Months of Injury, by Age Group and Low Fall, High Fall and Road Injury.

Age	Mortality within 12 months of injury (% mortality rate)	Trauma-related cause of death within 12 months of all blunt injury* (%)	Trauma-related Deaths after Low Fall** (%)	Trauma-related Deaths after Falls higher than 0.5m (%)	Trauma-related Deaths after Road Injuries (%)	P***
45–54	120 (9.35)	76 (63.3)	5 (18.5)	23 (82.1)	38 (95.0)	<0.001
55–64	211 (11.23)	103 (48.8)	22 (22.7)	14 (77.8)	55 (83.3)	<0.001
65–69	112 (14.66)	49 (43.8)	21 (32.8)	4 (66.7)	21 (75.0)	<0.001
70–74	123 (14.40)	33 (26.8)	13 (16.5)	5 (83.3)	15 (83.3)	<0.001
75–84	392 (19.70)	109 (27.8)	76 (24.4)	9 (42.9)	5 (78.3)	<0.001
Over 85	392 (29.25)	71 (18.1)	49 (14.8)	10 (38.5)	6 (60.0)	<0.001

*The most common non-trauma causes of death within 12 months of injury were: pneumonia (20.2%), cardiac (12.4%), cerebrovascular (12.3%), cancer (11.9%), respiratory (3.5%), urinary tract infections (2.7%) and renal (2.5%).

**The most common non-trauma causes of death after low falls were: pneumonia (23.8%), cerebrovascular (14.9%), cardiac (13.7%), cancer (13.7%), respiratory (3.5%), urinary tract infections (3.2%) and renal (3.2%).

***Fisher’s exact test

### Sensitivity analyses

The following checks were conducted to test the robustness of our result, with no effect on our findings: (i) Controlling for pattern of injury, adjusting for pattern of injury by Abbreviated Injury Scale (AIS) region with AIS score of 3 or more, and for polytrauma; (ii) Excluding non-residents (death registry captures 100% long-term death for residents); (iii) Different cut-off for defining low- versus high- falls: changing the low fall height definition to one metre, two metres and three metres showed a lower c-statistic than the initial definition of 0.5 m. Using same-level (as entered) versus any higher level showed similar c-statistics to low falls and similar effects; (iv) Addition of sub-categories of road injuries (pedestrian, motor vehicle, motorcycle)[29], the second commonest mechanism of injury after falls; (v) Separate sub-group analysis of moderate (ISS 9–15) and severe injuries (ISS>15); (vi) Regression using the entire adult population (age over 18), as in a recent study[14]; and (vii) Addition of clinically relevant 1–5% co-morbidities (see methods).

## Discussion

Our study shows that low fall patients were almost twice as likely to die within a year of injury compared to trauma patients with high fall heights and other blunt injuries, especially males, independent of injury severity. The cause of death in these cases was mostly not trauma-related.

The age-gender interaction after low falls adds an important dimension to modelling outcomes after trauma. Different authors have proposed different cut-offs for the effect of age on mortality after trauma[41, 43, 45]. In our study, there is excess mortality for males compared to females from age 70, and this excess mortality is mostly in patients with the low falls. Sub-group analyses by gender suggest that the effect of age on mortality begins at 55 for men and 70 years for women. Hence, there is no single age cut-off that would capture these interactions.

This may explain some of the conflicting findings in the literature on gender differences when low fall mechanism is not controlled for. In one study using matched-pair analysis, while males had a lower mortality rate, the proportion of falls below 3m for males was only 13% in the study population, versus 27% for females[47]. Both males and females in their study included some older patients, as the mean age was 43.5 and 52.1 years respectively. A study using American National Trauma Data Bank (NTDB) data showed females had fewer complications after injury[48], but while they controlled for mechanism of injury (falls), the height threshold to define high versus low fall in the NTDB is set at three metres.

Our study builds on recent literature showing poor outcomes after low falls[49, 50]. We found an effect in males at a younger age (55) than explored in these recent studies, controlling for region of injury, severity of injury and co-morbidities. Decreased upper and lower extremity strength are part of the clinical indicators of frailty[42, 51] and contribute to the mechanism of a low fall. We postulate that the low fall could be a surrogate marker of frailty and that frailty could explain the increased mortality in studies of fallers, even after controlling for age and injury severity. The increased mortality in males is similar to what has been reported on frailty and gender[52, 53]. The validity of this hypothesis should be further explored through future prospective studies which would examine other markers of frailty in patients after low falls compared to other trauma.

While our study was not designed to explore improvements in clinical outcomes, it is likely that patients who are frail are more likely to benefit from early comprehensive geriatric assessment and collaborative care, compared to non-frail older patients after trauma[54, 55]. Ideally, early comprehensive assessment should be performed for all older patients presenting with a fall, but for a busy geriatric service in a rapidly ageing population, our study suggests that priority be given to patients suffering significant injury after low falls compared to those suffering falls from a greater height, and that some of the conventional age cut-offs (65 or 70 being the commonest in our citations) may miss the younger males whose risk start at age 55. This can result in targeted interventions, better resource prioritization, and could explain the volume-outcome phenomenon specific to older trauma patients [16–18]. The low fall mechanism should be considered in the case mix when benchmarking trauma centres handling significant volumes of geriatric trauma[16–18].

While this is a national hospital-based cohort from an urban country in Asia, our study population appears fairly similar to other ageing populations. Our population life expectancy is long[56] and the overall fall incidence in our study is similar to that reported in a recent study from a state-wide trauma registry in North America with similar inclusion criteria[18]. All public hospitals have inpatient geriatric and orthogeriatrics services.

Our study has some limitations. First, injuries for which the SCDF ambulance was not called and were admitted to a private hospital would not enter the National Trauma Registry. This would have a minor effect on the capture of moderate injuries as SCDF usage is high[8, 57]. Secondly, injuries with ISS below 9 and isolated hip fractures in the over 65 sustained in same-level falls were excluded from our study as detailed physiological and co-morbidity data were not captured. Our findings are significant despite being an underestimate for the over 65, since the over-65 isolated same-level fall hip fracture patients could not be included in our study due to lack of co-morbidity data. While there are good arguments both for and against the inclusion of isolated hip fractures in trauma registries[54–56], outcomes after hip fractures have been well-studied, showing increased mortality for males despite a lower incidence[58–62], and high incidence of frailty[63, 64]. These are consistent with the findings in our study, and suggest our findings would be similar if we were to repeat this study in future to include isolated hip fractures sustained in same-level falls in the over 65. Third, functional outcomes were not considered in this study as some were not yet available at the time of analysis. This would require comparison to baseline pre-injury functional information for comparison (only post-injury function is in NTR), especially in the frail patients, which we will include in a future prospective study.

## Conclusion

The low fall mechanism of injury was found to predict long-term and non-trauma-related mortality, independent of co-morbidities and injury severity, especially in males, and as such may be a surrogate marker of frailty in trauma registry data. We recommend a low fall definition of 0.5 m or less to optimize interpretation of trauma registry data in older adults sustaining moderate and severe injury. This study provides evidence for a different mechanism for long term mortality after moderate and severe injury for same-level and low falls, and further work should be undertaken to explore how to improve outcomes for these patients.
